# Health Workforce Challenges: Key Findings From the Swiss Cohort of Healthcare Professionals and Informal Caregivers (SCOHPICA)

**DOI:** 10.3389/ijph.2024.1607419

**Published:** 2024-07-26

**Authors:** Vladimir Jolidon, Jonathan Jubin, Emilie Zuercher, Leonard Roth, Tania Carron, Annie Oulevey Bachmann, Ingrid Gilles, Isabelle Peytremann-Bridevaux

**Affiliations:** ^1^ Unisanté, University Center for Primary Care and Public Health, Department of Epidemiology and Health Systems, University of Lausanne, Lausanne, Switzerland; ^2^ La Source School of Nursing, HES–SO University of Applied Sciences and Arts Western Switzerland, Lausanne, Switzerland; ^3^ Lausanne University Hospital, Lausanne, Switzerland

**Keywords:** healthcare professionals, well-being, retention intentions, professional trajectories, cohort study

## Abstract

**Objectives:**

The Swiss Cohort of Healthcare Professionals and Informal Caregivers (SCOHPICA) was created to study the career trajectories, retention intentions, and wellbeing of healthcare professionals (HCPs), addressing challenges such as staff turnover, low job satisfaction and burnout.

**Methods:**

SCOHPICA is a prospective open cohort. An electronic questionnaire was used to collect data from HCPs across multiple healthcare settings in Switzerland, encompassing the intention to stay in the profession, wellbeing, and various organizational, psychosocial, occupational and sociodemographic determinants.

**Results:**

The first (2022) baseline sample included 1707 HCPs from over 20 professions. Notably, 13% did not intend to stay in their profession, with intermediate caregivers (24%), registered nurses (17%) and pharmacists (17%) reporting the highest rates. Pharmacists scored lowest in wellbeing. Across determinants, pharmacists, physicians, and registered nurses reported worse scores for workload and work-life balance. Nursing professions had lower scores in various determinants, including influence at work, staffing and resource adequacy, and possibilities for development.

**Conclusion:**

SCOHPICA will provide critical insights on HCPs’ work conditions and experiences, supporting health workforce monitoring and management, and informing policy-making to ensure high-quality healthcare delivery.

## Introduction

Health workforce issues are high in the agenda of European policymakers, due to the multiple challenges affecting the wellbeing of health professionals’ and the functioning of health systems [1, 2]. These challenges are partly driven by labor market failures, health emergencies, underinvestment in the sector, health and demographic trends, as well as the rise of noncommunicable diseases [3]. The COVID-19 pandemic has intensified the issues, leading to increased demand for health services, higher levels of stress and burnout among health workers, including higher risks to their health and safety due to inadequate working conditions [4, 5].

Healthcare professionals (HCPs) are pivotal to health systems. Ensuring a sufficient number of professionals, equitable distribution and providing adequate training and working conditions are essential for delivering accessible and high-quality care [6]. The World Health Organization (WHO) has outlined objectives for 2030 to enhance performance and investment in the health workforce, strengthen health institution capacities, and improve data collection on HCPs [2]. The Working for Health 2022–2030 Action Plan [1] addresses these objectives, emphasizing the urgent need for investment in health workers’ education, skills, employment, and protection, as put forward by the Seventy-fourth World Health Assembly Resolution [7].

Across Europe, the challenges confronting the health workforce have been acute, with the WHO reporting staff shortages driven by insufficient recruitment and retention of HCPs, unattractive working conditions, limited opportunities for ongoing professional development, and poor mental health among health workers [6]. In Switzerland, working conditions for HCPs have deteriorated, exacerbated by the COVID-19 pandemic [8–10]. Career departures are a significant concern, with approximately 12% of physicians and 16%–19% of nurses and intermediate caregivers leaving their professions [11]. Projections suggest that by 2029, there will be a need for 70,000 nurses to both replace the existing workforce and meet population healthcare needs [12]. However, coverage rates are predicted to fall below 80%, indicating a substantial workforce deficit [12]. Forecasts also predict shortages in various medical specialties, necessitating reliance on foreign workforce [13]. Addressing these challenges requires comprehensive data to inform strategic actions aimed at safeguarding the health workforce, ensuring the resilience of health systems, and promoting population health.

Health workforce research has shown that organizational (e.g., workload, work environment, recognition, leadership), psychosocial (e.g., cohesion and social support), psychological (e.g., stress, resilience) and sociodemographic (e.g., age, gender) determinants may affect HCPs’ wellbeing and intention to stay in the job/profession [14–18]. In Switzerland, studies have also investigated job stress, job satisfaction, burnout, and intention to stay in/leave the job/profession among HCPs [19–31]. However, Swiss and international studies have mostly focused on physicians or nurses, often within a limited scope of settings (mainly hospitals), leaving other healthcare professions understudied [6, 32]. As a recent review has stressed [18], only a few studies have addressed issues of wellbeing and retention intentions among allied health workforce. Additionally, most studies have used cross-sectional designs, which do not capture the longitudinal experience of HCPs. In sum, nationwide and longitudinal research spanning multiple healthcare sectors and professions has been limited. Such research is essential to grasp the determinants influencing HCPs’ career trajectories, wellbeing, and intentions to stay in their profession. As the WHO stressed, such paucity of data and research may hamper the adequate planning, monitoring, coordination and management of the health workforce [6].

This paper presents findings on the wellbeing, intention to stay in the profession, and the determinants of these outcomes, among HCPs in Switzerland. It is part of the Swiss Cohort of Healthcare Professionals and Informal Caregivers (SCOHPICA) project, which aims to gather comprehensive nationwide and longitudinal data on the professional trajectories, experiences and conditions of HCPs and informal caregivers (ICs). While the SCOHPICA project encompasses both HCPs and informal caregivers (ICs) in its longitudinal study, this paper concentrates on HCPs, using data from SCOHPICA’s first baseline survey.

## Methods

### Study Design, Population, and Data

This study draws on an analysis of the first 2022 baseline survey from the HCPs’ cohort of SCOHPICA. SCOHPICA is a national prospective open cohort study that collects data from all types of HCPs (e.g., general practitioners, specialist physicians, medical assistants, nurses, nurse aides, paramedics, psychologists, physiotherapists, dieticians, pharmacists, etc.) who work in direct contact with patients across different healthcare settings (e.g., hospitals, private practices, clinics, nursing homes, community services, etc.) in Switzerland, regardless of their employment status (i.e., whether self-employed or salaried). Students, retired HCPs, and those who were not working at the time of the baseline survey are not eligible to participate. The questionnaire was developed in the Swiss national languages, thereby individuals who cannot read French, German or Italian were not included in the study. SCOPICA’s study protocol details the specifics of the study design, recruitment process, ethical considerations and planned analyses [33].

SCOHPICA’s first baseline survey was conducted from 1 October 2022 to 31 January 2023. While the survey aimed to reach at least 1,500 HCPs for adequate measurement precision, it obtained responses from 1853 HCPs. Data collection was carried out through a self-administered electronic questionnaire, which was made available to HCPs on SCOHPICA website [34]. This questionnaire, comprising approximately 140 questions, was designed to be completed in around 30 min. Participants provided their informed consent before starting the questionnaire.

### Intention to Stay in the Profession and Wellbeing of Healthcare Professionals

Two main outcome variables of the SCOHPICA study were considered. The first variable was the HCPs’ intention to stay in their profession, assessed through the question, “If your working conditions/environment were to remain the same over the next few months, would you stay in your current profession?” Responses were measured on a 5-point scale, ranging from “No, not at all” to “Yes, absolutely.” The second variable was the wellbeing of HCPs, which was evaluated using the Flourish Index [35]. This index consists of 10 items, each rated on a 10-point scale spanning from 1 indicating a low wellbeing to 10 a high wellbeing. The index’s score range also spans from 1 (low wellbeing) to 10 (high wellbeing).

### Determinants of the Intention to Stay in the Profession and Wellbeing

SCOHPICA’s baseline questionnaire was designed to gather data on a range of determinants that may influence HCPs' wellbeing and intention to stay in the profession. The selection of these determinants was informed by preliminary literature reviews [17, 18], and consultations with SCOHPICA’s expert panel. Details on the instruments used for measuring these determinants can be found in SCOHPICA’s protocol [33].

In this paper, we focus on the determinants that showed acceptable internal consistency, as evaluated using Cronbach’s alpha. These determinants are detailed, along with the number of items and scales used, primarily using Likert scales, as follows:

- Workload: 5 items; 5-point scale from “Less than once a month/Never” to “Several times a day,” score range of 1–5.
- Control over Working Time: 4 items; 5-point scale from “Never/Hardly ever” to “Very often/Always,” score range of 0–100.
- Staffing and Resource Adequacy: 5 items; 4-point scale from “Strongly disagree” to “Strongly agree,” score range of 1–4.
- Possibilities for Development: 3 items; 5-point scale from “To a very large extent” to “To a very small extent,” score range of 0–100.
- Work-life Balance: 5 items; 4-point scale from “Yes, absolutely” to “No, not at all,” score range of 0–100.
- Leadership: 7 items; 5-point scale from “Never/Hardly ever” to “Very often/Always,” score range of 1–5.
- Influence at Work: 6 items; 5-point scale from “Never/Hardly ever” to “Very often/Always,” score range of 0–100.
- Sense of Community at Work: 3 items; 5-point scale from “Never/Hardly ever” to “Very often/Always”, score range of 0–100.
- Interprofessional Collaboration: 14 items; 5-point scale from “Strongly disagree” to “Strongly agree,” score range of 1–5.
- Recognition at Work: 12 items; 5-point scale from “Strongly disagree” to “Strongly agree,” score range of 1–5.
- Preparedness to Work Reality: 2 items were analyzed separately, 1) “Do you feel that, overall, your training has prepared you well for your professional activity?” 2) “In my work, I use the full extent of my practice,” 5-point scale from “Strongly disagree” to “Strongly agree,” score range of 1–5.
- Meaning of Work: 2 items; 5-point scale from “To a very large extent” to “To a very small extent”, score range of 0–100.
- Intolerance to Uncertainty: 6 items; 5-point scale from “Not at all my characteristic” to “Entirely my characteristic,” score range of 1–5.
- Burnout: 1 item; 5-point scale from “I do not have burnout symptoms” to “I feel completely burned out,” score range of 1–5.
- Self-rated Health: 1 item; 5-point scale from “Excellent” to “Poor,” score range of 1–5.
- Job Satisfaction: 1 item; 4-point scale from “Very unsatisfied” to “Very satisfied,” score range of 0–100.

All determinants reflect a positive experience or condition with higher scores, except for workload, burnout, and self-rated health, where higher scores indicate a negative experience or condition.

### Socioprofessional and Sociodemographic Variables

The baseline questionnaire gathered data on aspects related to HCPs’ work and occupation:

- Current profession: paramedic, physician, medical assistant, pharmacist, midwife, registered nurse, physiotherapist, etc.
- Occupational context: public hospital, private hospital, solo/two-physician practice, group practice, home care, nursing home, pharmacy, etc.
- Occupational sector: somatic care, home care, mental health, rehabilitation, long-term care, other.
- Country of training: open-ended answer.
- Numbers of years in the profession: number of years.
- Employment rate: from 0% to 100%.
- Hours worked per week: number of hours.
- Managerial responsibilities: yes, no.
- Monthly individual income (in CHF): below 2000, 2001–4,000, 4,001–6,000, 6,001–8,000, 8,001–10000, more than 10,000.

Sociodemographic data was also collected:

- Gender: man, woman, other, do not wish to answer.
- Age: <35 years, 35–44 years, 45–54 years, ≥55 years.
- Nationality: Swiss, Swiss and other nationality, foreign national.
- Marital/partnership status: single, cohabiting partner/registered partnership/married, separated/dissolved partnership/divorced, widowed.
- Children: yes, no.
- Informal caregiving: yes currently, yes in the past, no.
- Language: German, French, Italian.

### Statistical Analyses

We conducted descriptive analyses to summarize the characteristics of each variable in our study. We calculated the median and interquartile range (IQR) for the score of each determinant, both for the overall sample and for specific professional groups which had more than 50 participants. When the median score of a professional group differed from the other professions, we used a non-parametric equality-of-medians test to assess the difference in median scores. This test offers the advantage of not requiring the data to be normally distributed and is less sensitive to outliers compared to a t-test. In our analysis, *p*-values of 0.05 and smaller were reported. However, we only considered *p*-values of 0.01 or lower as statistically significant, to account for multiple testing and adopt a more conservative approach. This adjustment was made to ensure greater rigor and reliability in our findings.

For handling occasional missing data, we applied listwise deletion, which involved excluding the cases with missing responses from the analyses. For cases with missing responses in determinant items, we calculated the score using the mean of the items to which participants did respond. This was only applied if participants had answered more than 50% of the items within a determinant and at least two items within a determinant.

All statistical analyses were carried out using StataBE 18.

## Results

### Description of the Sample

The 2022 SCOHPICA baseline survey comprised 1707 HCPs, following data cleaning that excluded ineligible cases and those with partial answers. Sociodemographic characteristics of the survey participants are presented in Table 1. Women accounted for 78.1% of the participants. In terms of age distribution, 30.8% were under 35 years old, 48.9% were between 35 and 54 years old, and 20.4% were 55 years or older. Swiss nationals made up 82.2%, while foreign nationals constituted 17.8%. Regarding marital status, 56.1% of the respondents were married or cohabiting, 33.1% were single, and 10.1% were separated or divorced. 53.7% of the participants had children. Concerning informal caregiving, 23% were current caregivers, and 12.1% had been caregivers in the past. Most participants completed the questionnaire in French (50.5%) or German (43%), with a smaller proportion in Italian (6.5%).

**TABLE 1:** Sociodemographic characteristics of 1707 healthcare professionals, Swiss Cohort of Healthcare Professionals and Informal Caregivers baseline data 2022. (Switzerland, 2024).

Variable name	%
Gender (N = 1701)	
Women	78.1
Men	21.2
Other	0.1
Do not wish to answer	0.6
Age (N = 1,657)	
<35 years old	30.8
35–44 years old	26.1
45–54 years old	22.8
≥55 years old	20.4
Nationality (N = 1,698)	
Swiss	67.3
Swiss and other nationality	14.9
Foreign national	17.8
Marital status (N = 1,699)	
Single	33.1
Cohabiting partner, registered partnership, married	56.1
Separated, dissolved partnership, divorced	10.1
Widowed	0.7
Children (N = 1,698)	
Yes	53.7
No	46.3
Informal caregiving (N = 1,689)	
No	64.9
Yes, currently	23.0
Yes, in the past	12.1
Questionnaire language (N = 1707)	
French	50.5
German	43.0
Italian	6.5

Note: N = number of participants who answered to the item.

Socioprofessional characteristics of HCPs are presented in Table 2. HCPs from over 20 different professions responded to the survey, with ten professional categories having 50 or more participants, which included registered nurses as the largest group (32.4%), followed by physicians (12.4%), physiotherapists (9.1%), occupational therapists (5.3%), medical assistants (4.4%), pharmacists (4.2%), advanced practice nurses and paramedics (each at 3.6%), and dietitians and intermediate caregivers (3.4% and 3.2%, respectively).

**TABLE 2:** Socioprofessional characteristics of 1707 healthcare professionals, Swiss Cohort of Healthcare Professionals and Informal Caregivers baseline data 2022 (Switzerland, 2024).

Variable name	%
Profession (N = 1705)	
Registered nurse	32.4
Physician	12.4
Physiotherapist	9.1
Occupational therapist	5.3
Medical assistant	4.4
Pharmacist	4.2
Paramedic	3.6
Advanced practice nurse	3.6
Dietitian	3.4
Intermediate caregiver	3.2
Pharmacy assistant	2.7
Midwife	2.7
Auxiliary caregiver	2.1
Specialized nurse	1.7
Speech therapist	1.5
Osteopath	1.5
Psychologist-psychotherapist	1.5
Other	1.1
Psychologist	0.8
Radiology technologist	0.6
Specialist caregiver	0.4
Chiropractor	0.4
Complementary therapist	0.4
Surgical technologist	0.2
Ambulance technician	0.2
Pharmacy operations assistant	0.2
Dentist	0.1
Podologist	0.1
Dental assistant	0.1
Occupational context^a^ (N = 1,699)	
Public hospital	34.1
Group practice	14.6
Solo/two-person practice	12.8
Home care	11.7
Nursing home	8.9
Pharmacy	7.1
Private hospital/clinic	6.2
Emergency/rapid response/ambulance	5.5
Other	5.5
Rehabilitation treatment centre	4.4
School environment	3.2
Enterprise	1.9
Institution for individuals requiring assistance (other than nursing homes)	1.5
Health centre	1.2
NGO/associations/foundations	0.9
Medical laboratory	0.1
Occupational sector^a^ (N = 1,679)	
Somatic care	60.8
Home care	20.2
Mental health	19.5
Rehabilitation	18.5
Long-term care	17.9
Other	11.6
Country of training (N = 1,697)	
Switzerland	81.6
Switzerland and other country	1.2
Europe	16.7
Outside Europe	0.5
Number of years in the profession (N = 1700)	
<5 years	17.9
5–14 years	36.0
15–24 years	21.8
>25 years	24.4
Employment rate (N = 1,618)	
<50%	6.4
50%–89%	47.5
90%–100%	46.1
Number of hours worked per week (N = 1,696)	
<20 h/week	6.8
20–29 h/week	14.2
30–39 h/week	26.0
40–49 h/week	37.6
>50 h/week	15.5
Managerial responsibility (N = 1,695)	
Yes	29.9
No	70.1
Monthly income (N = 1,695)	
<2,000 CHF	3.5
2,001–4,000 CHF	21.8
4,001–6,000 CHF	41.6
6,001–8,000 CHF	19.2
8,001–10,000 CHF	6.9
>10,000 CHF	7.0

Notes: N = number of participants who answered to the item.

^a^ Multiple answers allowed.

Regarding the occupational context, 34.1% of the participants worked in public hospitals, followed by 14.6% in group practices and 12.8% in solo or two-person practices. The majority of respondents were involved in somatic care (60.8%), followed by home care (20.2%), mental health (19.5%), rehabilitation (18.5%), and long-term care (17.9%). Most HCPs received their training in Switzerland (82.8%), with a smaller proportion trained in Europe (16.7%). In terms of experience, 36% had 5–14 years of experience, 21.8% had 15–24 years, and 24.4% had over 25 years. Work hours varied, with 37.6% working 40–49 h per week, 26% working 30–39 h, and 15.5% working more than 50 h. Managerial responsibility was held by 29.9% of the participants, and the largest group (41.6%) earned 4,001–6,000 CHF per month.

### Intention to Stay in the Profession and Wellbeing of Healthcare Professionals

**FIGURE 1:**
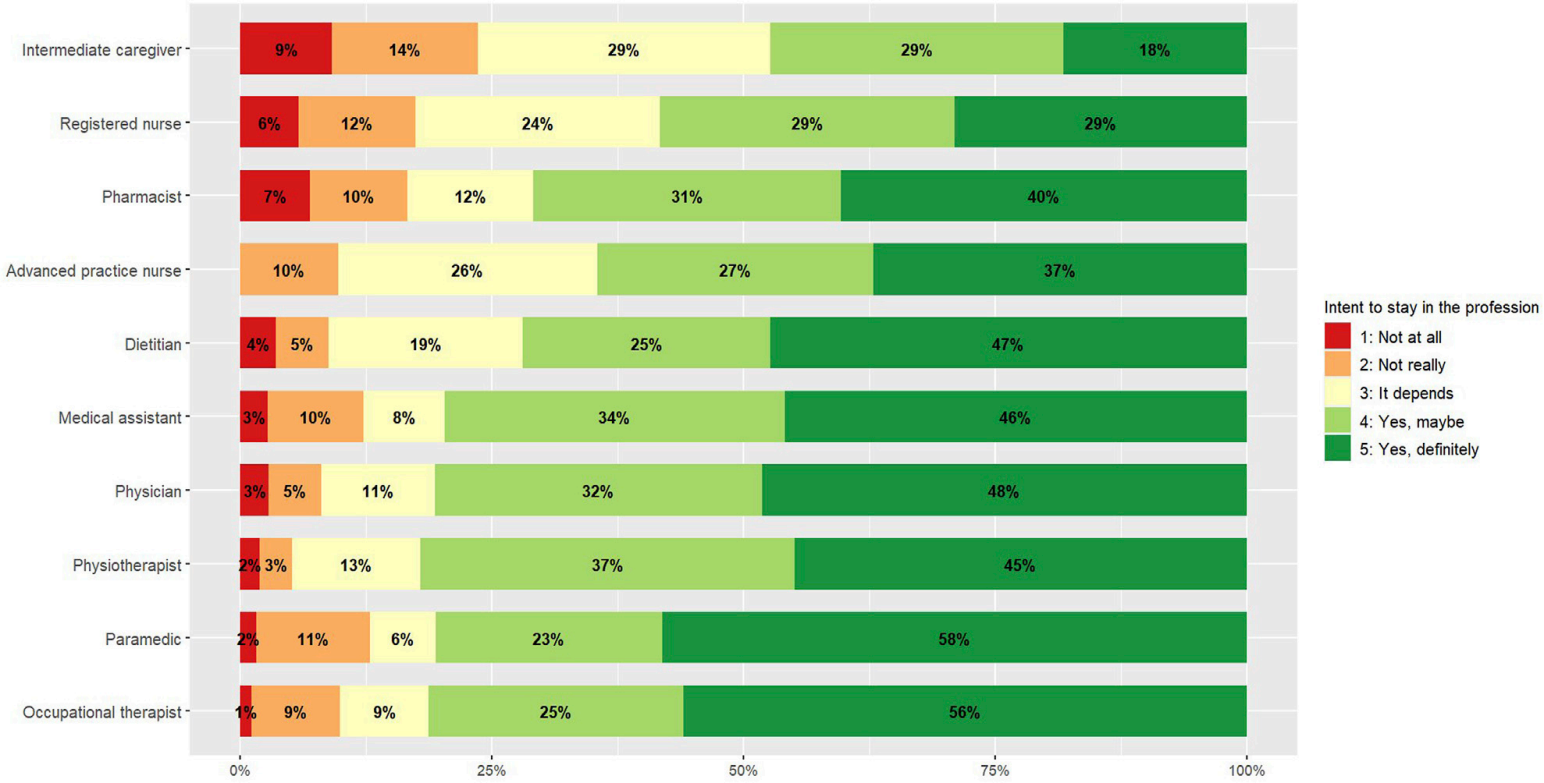
Intention to stay in the profession, by profession (Switzerland, 2024). Note: Results are presented only for professional categories with ≥50 participants.

**FIGURE 2:**
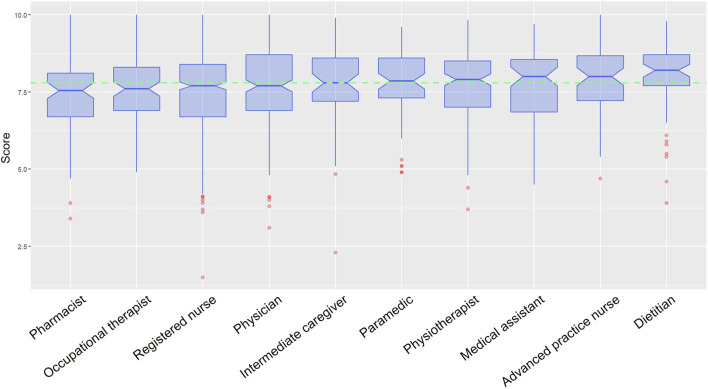
Wellbeing (Flourish index), by profession (Switzerland, 2024). Note: Results are presented only for professional categories with ≥50 participants.

Figure 1 illustrates HCPs’ intention to stay in their profession. Overall, 13.2% of HCPs did not intend to stay in their profession when combining the responses of “no, not at all” and “no, not really.” This rate was highest among intermediate caregivers (23%), registered nurses (18%), pharmacists (17%), paramedics (13%) and medical assistants (13%). Regarding wellbeing, as measured by the Flourish Index, the overall score was 7.8 (Table 3). Pharmacists reported the lowest wellbeing median score at 7.5, followed by occupational therapists (7.6), registered nurses (7.7) and physicians (7.7), although their scores were not significantly lower than the other professions. Figure 2 depicts HCPs’ wellbeing scores by profession.

**TABLE 3:** Healthcare professionals' wellbeing, intention to stay in the profession and determinants, by profession, median and interquartile range (IQR) (Switzerland, 2024).

	Overall score (n = 1707)	Intermediate caregivers (n = 55)	Medical assistant (n = 75)	Registered nurse (n = 553)	Dietitian (n = 58)	Pharmacist (n = 72)	Advanced practice nurse (n = 62)	Occupational therapist (n = 91)	Physiotherapist (n = 156)	Physician (n = 212)	Paramedic (n = 62)
	Median (IQR)	Median (IQR)	Median (IQR)	Median (IQR)	Median (IQR)	Median (IQR)	Median (IQR)	Median (IQR)	Median (IQR)	Median (IQR)	Median (IQR)
Intent to stay in the profession	4.0 (2.0)	3.0 (1.0) **	4.0 (1.0)	4.0 (2.0)	4.0 (2.0)	4.0 (2.0)	4.0 (2.0)	5.0 (1.0) **	4.0 (1.0)	4.0 (1.0)	5.0 (1.0) **
Wellbeing	7.8 (1.6)	7.8 (1.5)	8.0 (1.8)	7.7 (1.7)	8.2 (1.0) **	7.5 (1.4)	8.0 (1.5)	7.6 (1.4)	7.9 (1.5)	7.7 (1.8)	7.9 (1.3)
*Determinants*											
Burnout	2.0 (1.0)	2.0 (1.0)	2.0 (0.0)	2.0 (1.0)	2.0 (1.0)	2.0 (1.0)	2.0 (1.0)	2.0 (1.0)	2.0 (1.0)	2.0 (1.0)	2.0 (1.0)
Control over working time	50.0 (25.0)	37.5 (18.8) **	56.3 (25.0)*	43.8 (31.3)*	65.6 (25.0) ***	50.0 (25.0)	59.4 (25.0) ***	50.0 (31.3)	43.8 (25.0)*	50.0 (31.3)	50.0 (31.3)
Interprofessional collaboration	3.6 (0.9)	3.6 (1.1)	4.0 (0.4) ***	3.5 (0.9)*	3.6 (0.6)	3.1 (0.7) **	3.5 (0.7)	3.6 (0.7)	3.4 (0.9) **	3.8 (0.9) ***	3.8 (1.0)
Influence at work	54.2 (33.3)	37.5 (29.2) ***	54.2 (25.0)	41.7 (29.2) ***	60.4 (33.3)*	56.3 (33.3)	54.2 (20.8)	66.7 (33.3) ***	66.7 (33.3) ***	62.5 (29.2) ***	50.0 (33.3)
Intolerance to uncertainty	2.3 (1.2)	2.3 (1.2)	2.5 (1.0)	2.3 (0.8)	2.3 (0.8)	2.7 (1.2) **	2.5 (1.0)	2.3 (1.0)	2.5 (1.0)	2.5 (1.1)	1.9 (0.7) ***
Job satisfaction	67.0 (34.0)	67.0 (34.0)	67.0 (0.0)	67.0 (34.0)	67.0 (33.0)	67.0 (17.0)	67.0 (34.0)	67.0 (0.0)	67.0 (0.0)	67.0 (0.0)	67.0 (33.0)
Leadership	3.6 (1.6)	3.6 (1.7)	3.9 (1.3)	3.6 (1.4)	3.9 (1.0)	3.4 (1.6)	3.7 (1.4)	3.7 (1.4)	3.6 (1.7)	3.6 (1.6)	3.6 (1.3)
Meaning of work	87.5 (25.0)	100.0 (25.0)	87.5 (25.0)	87.5 (25.0)	75.0 (12.5) **	100.0 (25.0)*	87.5 (25.0)	87.5 (25.0)	87.5 (25.0)	87.5 (25.0)	87.5 (25.0)
Possibilities for development	66.7 (25.0)	58.3 (25.0) **	66.7 (25.0)	66.7 (25.0) ***	66.7 (16.7)	66.7 (20.8)	66.7 (25.0)	75.0 (25.0) **	75.0 (25.0)*	75.0 (29.2) **	75.0 (25.0)
Preparedness to work reality 1^a^	4.0 (1.0)	4.0 (1.0)	4.0 (1.0)	4.0 (1.0)	4.0 (0.0)	4.0 (2.0)	4.0 (0.0)	3.0 (2.0)	4.0 (0.0)	4.0 (1.0)	4.0 (0.0)
Preparedness to work reality 2^a^	4.0 (2.0)	3.0 (2.0)	4.0 (2.0)	4.0 (2.0)	3.0 (2.0)	4.0 (2.0)	3.5 (2.0)	3.0 (2.0)	4.0 (1.0)	4.0 (1.0)	4.0 (2.0)
Recognition at work	3.5 (1.0)	3.4 (1.0)	3.9 (1.0)***	3.5 (1.1)	3.3 (0.9)	3.4 (1.1)	3.6 (0.9)	3.7 (0.9)	3.4 (1.0)	3.5 (1.0)	3.6 (0.8)
Self-rated health	3.0 (1.0)	3.0 (1.0)	3.0 (1.0)	3.0 (1.0)	2.0 (1.0)	3.0 (1.0)	3.0 (1.0)	3.0 (1.0)	3.0 (1.0)	2.0 (1.0)	2.0 (1.0)
Sense of community at work	75.0 (25.0)	75.0 (25.0)	91.7 (25.0) ***	75.0 (25.0)	75.0 (25.0)	75.0 (25.0)	75.0 (16.7)	83.3 (25.0) **	83.3 (25.0)	75.0 (25.0)	83.3 (16.7)
Staffing and resource adequacy	2.4 (1.0)	2.2 (1.2)	2.8 (0.8) **	2.4 (1.0) ***	2.8 (0.8)	2.6 (0.8)	2.2 (0.8) **	2.8 (0.8)	2.6 (0.8)	2.4 (0.6)	3.0 (0.6) ***
Work-life balance	53.2 (40.2)	53.4 (40.4)	73.6 (27.0) ***	46.6 (33.6) ***	67.0 (27.0) **	43.3 (40.4)	46.8 (39.8)	66.6 (40.2) ***	56.7 (40.0)	33.2 (33.6) ***	66.8 (27.0) ***
Workload	3.8 (1.8)	3.4 (2.0)	3.8 (1.6)	4.0 (1.6) ***	3.2 (1.8)	4.2 (1.2) **	3.8 (1.4)	2.8 (1.6) ***	3.4 (1.8)	4.2 (1.4) ***	3.1 (1.1) ***

Notes: Results are presented only for professional categories with ≥50 participants. When the median score of a professional group differed from that of other the professional groups, a non-parametric test for equality of medians was used to assess the significance of the difference, with significance levels indicated as: *p-value ≤0.05, **p-value ≤0.01, ***p-value ≤0.001.

^a^ Preparedness to work reality: 1) “Do you feel that, overall, your training has prepared you well for your professional activity?” and 2) “In my work, I use the full extent of my practice”.

### Determinants of the Intention to Stay in the Profession and Wellbeing

Table 3 presents the results of the determinants of HCPs’ intention to stay in the profession and wellbeing. The median score for workload was 3.8, with registered nurses, pharmacists, and physicians experiencing significantly higher workloads than the median of other professions. Compared to other professional groups, registered nurses reported significantly lower scores in staffing and resource adequacy, possibilities for development, work-life balance and influence at work. Physicians also had a significantly lower work-life balance score. Physiotherapists and pharmacists reported lower scores in interprofessional collaboration. Advanced practice nurses scored lower in staffing and resource adequacy, paramedics in intolerance to uncertainty, dietitians in meaning of work, and intermediate caregivers in control over working time, possibilities for development and influence at work. Medical assistants and occupational therapists, in contrast, did not exhibit lower scores in any determinant compared to other professional groups. Finally, no significant differences were observed between professional groups in terms of leadership, job satisfaction and self-rated health scores.

## Discussion

This paper presented findings on the wellbeing and intention to stay in the profession of HCPs, along with the determinants of these outcomes, based on data from 1707 HCPs in SCOHPICA’s first baseline survey (2022). Notably, the data collection spanned over 20 healthcare professions across various healthcare settings throughout Switzerland. The results indicated that a concerning proportion of HCPs (13%) did not intend to stay in their profession should their current working conditions persist. This figure was highest among intermediate caregivers (23%), registered nurses (18%) and pharmacists (17%). Furthermore, the results highlighted divergences between professions across different factors. Registered nurses, physicians, and pharmacists reported the highest workload levels and lower work-life balance scores. Nursing professions (registered nurses, advanced practice nurses and intermediate caregivers) faced common challenges related to staffing and resource adequacy, influence at work, opportunities for development and control over working time.

The high turnover intentions observed in our study among intermediate caregivers and registered nurses align with findings from a Swiss study, which reported an 18.5% intention to leave the profession among caregivers and nurses [27]. Similarly, another study spanning four European countries found that 13.6% of nurses expressed the intention to leave their profession [36]. However, accurately comparing the prevalence of turnover intentions across different studies is challenging due to variations in definitions and measurements. Some studies may focus on intentions to stay in or leave the job, while others focus on staying in/leaving the profession. The timeframe used to assess these intentions also varies, with some studies evaluating turnover intentions prospectively over the next several years and others retrospectively, considering intentions within the past year. Some studies do not specify any timeframe at all. In our study, we focused on the intention to stay in the profession, considering the near future (i.e., “the next few months”) as the timeframe, as in the studies by Maniscalco and colleagues [36] and Hammig [27]. Surveying healthcare professionals (HCPs) who have already left the profession would ideally provide better insights into their turnover intentions. However, recruiting these individuals presents significant challenges and feasibility issues.

This study identified several determinants that disproportionately affected certain professions. Previous research has stressed the significant association of these determinants with intentions to leave those professions. For instance, we observed that physicians experienced workload and work-life balance issues, and pharmacists grappled with workload and lower wellbeing, while previous studies highlighted these determinants as significant drivers of turnover intentions among physicians and pharmacists [18, 24, 27, 29, 37, 38]. Additionally, nursing professions faced challenges related to workload, work-life balance, staffing and resource adequacy, influence at work, opportunities for development and control over working time, all of which were shown to contribute to nurses leaving their profession in past research [20, 23, 27, 29]. Notably, our results revealed that both intermediate caregivers and registered nurses (the two professions with the lowest intention to stay in the profession) had the lowest scores in control over working time, influence at work and possibilities for development, compared to other professions, characterizing these two professions.

Practical implications of our results involve the need for targeted interventions to improve working conditions, reduce turnover intentions, and enhance wellbeing among HCPs, which are relevant both in the Swiss context and internationally. Importantly, our study highlights the necessity of adopting a comparative perspective that considers multiple healthcare professions and their unique challenges. Acknowledging the differences and commonalities between professions will enable the development of appropriate strategies. Hence, future research, policymakers and healthcare managers should identify and prioritize the key factors influencing turnover intentions across different professions. Additionally, monitoring the impact of these interventions on HCP’s turnover intentions and wellbeing in future research will help inform and refine retention strategies.

Specific recommendations include enhancing working conditions by implementing measures to manage and reduce workload, particularly for physicians, pharmacists, and nurses. This involves promoting initiatives that support work-life balance and provide access to staff support services and resources. Increasing HCPs’ control over their schedules is essential, allowing for more autonomy and flexibility to accommodate personal needs and reduce burnout. Professional development and career growth should be fostered by developing clear career paths and providing opportunities for continuing education and mentoring programs. Retention strategies should be developed for professions with high turnover intentions, such as intermediate caregivers and registered nurses. These strategies should aim at increasing empowerment in the workplace and may include recognition and reward systems, flexible scheduling, enhanced autonomy, and clear pathways for career progression. Additionally, conducting regular constructive feedback sessions can allow for adjustments in working conditions, reduce turnover, and provide insights to inform retention strategies.

Interestingly, our study found that 18% of HCPs were foreign nationals, with an equal percentage having received their training abroad. This underscores the reliance on international healthcare workers and their importance to the Swiss healthcare system, as pointed out in previous reports [12, 13]. Health workforce migration is an international challenge affecting many countries, as highlighted in a recent WHO report [6], and is strongly related to the issue of health workforce shortages. Given the crucial role of these migration movements, our future research will examine these issues in greater detail, exploring their relationship to HCPs’ intentions to stay in the profession and their wellbeing.

This is the first study providing insights into HCPs’ intention to stay in their professions, their wellbeing, and the determinants thereof, across multiple healthcare professions and settings throughout an entire country. Previous research lacked this breadth of coverage, hindering comparisons of diverse experiences and conditions among HCPs. Such comprehensive information is highly valuable for healthcare stakeholders, especially given the lack of accurate and thorough data on HCPs, and the highlighted workforce shortages within the Swiss healthcare system [12, 13]. Hence, SCOHPICA has the potential to play a crucial role in monitoring the conditions of healthcare workers, thereby supporting the design of management and policy interventions aimed at improving working conditions and retaining HCPs. This aligns with international recommendations to enhance data collection on the health workforce, not only to address pressing challenges such as staff shortages but also to effectively plan, manage, coordinate and inform decisions concerning the health workforce [1, 2, 6].

This study is part of the broader SCOHPICA project framework, which employs a cohort design that will be complemented by life history calendars. Subsequent investigations within this project will delve into the professional trajectories of HCPs using longitudinal analysis, as well as optimal matching and clustering techniques to create typologies of professional trajectories. A longitudinal perspective will be important for understanding the transition from intention to the actual decision to stay in/leave the profession. Additionally, future analyses will apply advanced statistical methodologies, including structural equation modeling, to elucidate the intricate relationships and mediating pathways among the determinants affecting HCPs’ professional trajectories, retention intentions, and wellbeing.

It is important to acknowledge the limitations of this study. Firstly, due to the impracticality of drawing representative samples of all HCPs and obtaining their contact emails in Switzerland, the study employed non-probability sampling. Secondly, certain professional categories may have been underrepresented, particularly those that are more challenging to recruit, and smaller sample sizes might have influenced the ability to detect statistically significant differences. However, we anticipate increased participation from a diverse range of professions in future data collection waves. In fact, as an open cohort, SCOHPICA will recruit new participants annually, thereby increasing the cohort size, enhancing statistical power and representativeness, and enabling subgroup and stratified analyses. For upcoming survey waves, the SCOHPICA team will continue to collaborate closely with professional associations of HCPs at both national and regional levels, and with employers of HCPs (e.g., hospitals, home care, etc.). This should promote participation in the survey, particularly among professions that were underrepresented in the first survey wave. To reach HCPs, we employ targeted communication and recruitment strategies. For example, we provide communication packages to associations, enabling them to share the SCOHPICA questionnaire link with their members via emails, newsletters, and websites. Thirdly, there is a risk of selection bias if individuals who choose not to respond to the survey differ significantly in their characteristics from those who do participate. Without data on non-respondents, we are not able to assess the extent of this bias. Lastly, this study relies on self-reported data, which can be subject to recall and social desirability biases, leading to potential measurement bias. To mitigate these biases, the SCOHPICA questionnaire incorporated validated questions and underwent pre-testing to ensure its reliability and accuracy.

To conclude, the comprehensive scope of SCOHPICA’s study fills a significant gap in existing research, covering all healthcare professions across different sectors and providing critical insights into the health workforce. With the scarcity of data on HCPs and the pressing need for improved workforce planning, SCOHPICA’s findings will be crucial for addressing challenges such as attrition, staff shortages, inadequate working conditions, increased workloads, and burnout in the healthcare workforce. By supporting the monitoring, planning, and management of Switzerland’s health workforce, SCOHPICA will play a key role in addressing health system challenges, informing future policies, implementing targeted interventions, and promoting the delivery of high-quality care.
